# Sequence mining and transcript profiling to explore differentially expressed genes associated with lipid biosynthesis during soybean seed development

**DOI:** 10.1186/1471-2229-12-122

**Published:** 2012-07-31

**Authors:** Huan Chen, Fa-Wei Wang, Yuan-Yuan Dong, Nan Wang, Ye-Peng Sun, Xiao-Yan Li, Liang Liu, Xiu-Duo Fan, Hai-Long Yin, Yuan-Yuan Jing, Xin-Yue Zhang, Yu-Lin Li, Guang Chen, Hai-Yan Li

**Affiliations:** 1 Ministry of Education Engineering Research Center of Bioreactor and Pharmaceutical Development, Jilin Agricultural University, Changchun, Jilin, 130118, China; 2 College of Life Sciences, Jilin Agricultural University, Changchun, Jilin, 130118, China

**Keywords:** Gene expression, Lipid, RNA-sequencing, Soybean, Unigene

## Abstract

**Background:**

Soybean (*Glycine max* L.) is one of the most important oil crops in the world. It is desirable to increase oil yields from soybean, and so this has been a major goal of oilseed engineering. However, it is still uncertain how many genes and which genes are involved in lipid biosynthesis.

**Results:**

Here, we evaluated changes in gene expression over the course of seed development using Illumina (formerly Solexa) RNA-sequencing. Tissues at 15 days after flowering (DAF) served as the control, and a total of 11592, 16594, and 16255 differentially expressed unigenes were identified at 35, 55, and 65 DAF, respectively. Gene Ontology analyses detected 113 co-expressed unigenes associated with lipid biosynthesis. Of these, 15 showed significant changes in expression levels (log_2_fold values ≥ 1) during seed development. Pathway analysis revealed 24 co-expressed transcripts involved in lipid biosynthesis and fatty acid biosynthesis pathways. We selected 12 differentially expressed genes and analyzed their expressions using qRT-PCR. The results were consistent with those obtained from Solexa sequencing.

**Conclusion:**

These results provide a comprehensive molecular biology background for research on soybean seed development, particularly with respect to the process of oil accumulation. All of the genes identified in our research have significance for breeding soybeans with increased oil contents.

## Background

Soybean [*Glycine max* (L.) Merrill], one of the most important oil crops in the world that accounts for less than a third (27.7%) of the total global vegetable oil production. At present, the main producers of soybean are the United States, Brazil, Argentina, India, and China. Although world soybean oil production has steadily increased, rising from 37.73 million metric tons in 2007 to 43.65 million metric tons in 2012 (http://www.fas.usda.gov/psdonline/psdreport.aspx?hidReportRetrievalName=BVS&hidReportRetrievalID=708&hidReportRetrievalTemplateID=8), the production of soybean oil is still insufficient to meet the consumer demand, and the low oil content in soybean seeds is the main factor restricting yield. Therefore, an increase in soybean oil yield is desirable and has been one of the most major goals of oilseed engineering.

To increase the production of soybean oil, it is not feasible to extend the planting area of the crop, because of increased competition for land by the rapidly rising population. Therefore, it is a better strategy to increase the seed oil content than to increase the planting area [1]. In recent years, molecular genetics approaches have been used to modify seed oil content. Over-expression of a diacylglycerol acyltransferase (AtDGAT) cDNA in wild-type *A. thaliana* enhanced oil deposition and average seed weight [2]. The research of Wang *et al.* indicated that the oil content of soybean seeds could be increased by upregulation of two soybean Dof-type transcription factor (GmDof) genes, which are associated with fatty acid biosynthesis [3]. The soybean genome also has been examined with regard to the relationships between gene expression profiles and gene function. Although the biochemical pathways that produce different storage components are well characterized, there is still no integrated model describing differentially expressed genes (DEGs) involved in soybean lipid biosynthesis. With the development of high-throughput technologies, including the newly developed Solexa/Illumina RNA-sequencing and DEG high-throughput deep sequencing approaches, new genes have been discovered and specific transcripts analyzed. In Severin’s research, RNA Seq-Atlas provided a record of high-resolution gene expression in a set of various tissues. They also found dramatic tissue-specific gene expression of both the most highly-expressed genes and the genes specific to legumes in seed development and nodule tissues [4]. Furthermore, these technologies are useful for estimating overall gene expressions at different development stages and/or in different tissues, such as on rice transcriptome and disease [5-7].

In this study, we used Illumina Solexa sequencing to investigate gene expression in soybean seeds at different developmental stages (15, 35, 55, and 65 days after flowering; DAF), and then compared transcript reads with the most recent release of the *G. max* genome sequence (assembly Glyma1.01). Illumina Solexa *de novo* sequencing technology identified a total of 11592, 16594, and 16255 differentially expressed unigenes between the 15 and 35 DAF seeds, the 15 and 55 DAF seeds, and the 15 and 65 DAF seeds, respectively (Additional file 1: Tables S1, Additional file 2: Table S2, Additional file 3: Table: S3). Of these, 9905 unigenes existed in all three of these contrast groups and represented co-expressed unigenes. Furthermore, 124 candidate unigenes were screened from the three contrasting cDNA libraries and characterized as those responsible for lipid synthesis. Our researches provide the whole picture of DEG transcription patterns and expression levels during soybean seed development. The elucidation of DEG transcription patterns at specific stages of seed development also lays the foundation for understanding the molecular mechanisms underlying oil production. This research provides direction for controlling genes over-expression to increase soybean oil content.

## Methods

### Plant culture and collection

The soybean cultivar Jiyu-72 was used as the experimental material. The plants were grown in a green house with a 15-h light (200 μEm^-2^ s^-1^, 25°C)/9-h dark photoperiod (23°C), with relative humidity controlled at 75%. The developmental processes of soybean seeds from flowering to seed maturity were observed from July to October 2010. Pods were harvested at 15 DAF (immature stage), and then every 5 days until 70 DAF (pods containing mature seeds). After removing the seed coat, the seeds were used for oil extraction or frozen at −80°C until mRNA extraction and sequencing.

### Measurement of oil content

To extract the oil (or lipid), seeds harvested at 15, 20, 25, 30, 35, 40, 45, 50, 55, 60, 65, and 70 DAF were oven-dried to constant weight at 85°C. The dry samples were ground to a fine powder in a disintegrator, and the powder was transferred into 10-mL glass tubes for oil extraction. Oil was extracted using ligarine to determine total lipids (TL) gravimetrically [8]. Oil extraction was performed using a SER148 3/6 Extraction apparatus (VELP Scientifica, Italy). Extractions were carried out using triplicate samples for each stage, and mean values were determined. Errors are shown as standard deviations.

### Total RNA extraction, library construction, and deep sequencing

RNA was isolated from the seeds harvested at 15, 35, 55, and 65 DAF, respectively, using a TIANGEN RNA Prep Pure Plant kit (Tiangen Biotech Co. Ltd., Beijing, China) following the manufacturer’s protocol, followed by a chloroform extraction to improve RNA purity. The yield and quality of total RNA samples were determined using a ND-1000 NanoDrop spectrophotometer. For RNA library construction and deep sequencing, equal quantities of RNA samples from seeds at the four developmental stages were pooled. Approximately 6 μg RNA representing each group was submitted to Solexa (now Illumina Inc.) for sequencing.

### Differential expression (DE) detection

The raw data were filtered to remove adaptor reads, low quality reads, and reads of copy number = 1, yielding a dataset consisting of clean reads. For the tissue-specific analyses, raw digital gene expression data were normalized using a variation of the *reads*/Kb/million (RPKM) method [9,10]. The RPKM method corrects for biases in total gene exon size and normalizes for the total short read sequences obtained in each tissue library. Genes with data P-value <0.005, false discovery rate (FDR) ≤0.001, and estimated absolute log_2_-fold change ≥1 in sequence counts across libraries were considered to be significantly differentially expressed. Differentially expressed genes that were co-expressed during these four stages were then subjected to cluster analysis using the R program (a language and environment for statistical computing and graphics). Functional categorization of stress-regulated genes was performed using BGI WEGO (http://wego.genomics.org.cn/cgi-bin/wego/index.pl).

### Quantitative real-time PCR (qRT-PCR) analysis

To verify the data obtained by Solexa RNA-seq, qRT-PCR was performed on 12 genes with log_2_ratios ranging from 2 to 11 (Additional file 4: Table S4). The RNA samples used for the qRT-PCR assays were the same as those used for the DEG experiments. qRT-PCR was performed on a Mx3000P instrument (Stratagene), with SYBR-Green detection (Quant qRT-PCR Kit, Tiangen Biotechnology), according to the manufacturer's instructions. The soybean tubulin gene was used as an internal control (forward primer: 5’-ATGTTCCGTGGCAAGATGAG-3’, reverse primer: 5’-CATTGTTGGGAATCCACTC-3’) [11]. Primers were designed using the Primer Premier 5.0 and Oligo 6 programs to amplify products approximately 150-bp long. Thermal cycle conditions were as follows: 2 min at 95°C followed by 40 cycles of 15 s at 95°C, 15 s at 56-57°C, and 15 s at 72°C. Each cDNA was analyzed three times, after which the average threshold cycle (Ct) was calculated per sample. Standard curves were established for all investigated genes using a series of amplicon dilutions. The relative expression levels were calculated as 2^- (ΔCt of 35, 55, 65 DAF -ΔCt of 15 DAF).^

## Results

### Accumulation of oil at different stages of seed development

**Figure 1:**
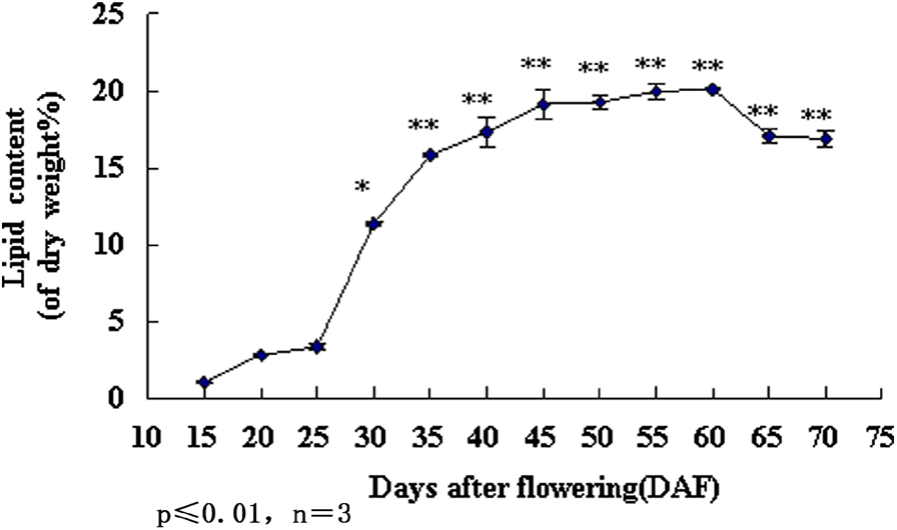
** Changes in lipid content during seed development.** Lipid content was determined every 5 days. One star stands for the discrepancy is significant and double stars stand for the discrepancy is extremely significant.

To explore lipid accumulation during development of soybean seeds, we quantified lipid content in soybean seeds harvested from 15 to 70 DAF. As shown in Figure 1, the oil content began to increase from 15 DAF, then markedly increased from 15 to 20 DAF, and then remained stable from 20 to 25 DAF before again increasing rapidly from 25 to 35 DAF. From 35 to 55 DAF, the oil content showed a steady increase, peaking at 60 DAF before dropping at 65 DAF. These results indicated that for the soybean cultivar JiYu-72, 15 DAF marked the beginning of oil accumulation, 35 DAF was the key stage for the rapid increase in the oil content. 55 DAF was the stage at which oil content was high and stable, and 65 DAF was the stage at which oil content decreased and nearly unchanged. Therefore, in order to explore the differentially expressed genes associated with lipid biosynthesis during soybean seed development, the following sequencing and qRT-PCR analyses were performed using samples from these four different developmental stages.

### Screening of differentially expressed genes (DEGs) from massive datasets

Initially, Solexa RNA-sequencing revealed a total of 11592, 16594, 16255 differentially expressed unigenes between 35 DAF and 15 DAF seeds, 55 DAF and 15 DAF seeds, and 65 DAF and 15 DAF seeds, respectively (Table 1). Among them, 9905 unigenes were present in all three of these contrast groups, and 1687, 6689, and 6350 were differentially expressed only in the 35 vs 15 DAF, 55 vs 15 DAF, and 65 vs 15 DAF groups, respectively. From the large datasets, we found that down-regulated genes were more abundant than up-regulated genes during soybean seed development. The normalized data are shown as scatter plots of log_10_-transformed transcription counts for the four different samples (Additional file 5: Figure S1).

**Table 1:** Differentially expressed genes between seeds of 35 DAF and 15 DAF, 55 DAF and 15 DAF, and 65 DAF and 15 DAF

**Contrast groups**	**No. of genes**						
	**Total genes**	**Co-expressed genes**	**Distinct genes**	**Up-regulated**		**Down-regulated**	
				**Known**^**a**^	**Unknown**^**b**^	**Known**^**a**^	**Unknown**^**b**^
**Seeds of 35DAF-VS-15DAF**	**11592**	**9905**	**1687**	**419**	**261**	**7314**	**3598**
**Seeds of 55DAF-VS-15DAF**	**16594**	**9905**	**6689**	**646**	**358**	**10203**	**5378**
**Seeds of 65DAF-VS-15DAF**	**16255**	**9905**	**6350**	**529**	**321**	**10246**	**5159**

‘known^a^’ represents genes with Gene Ontology annotation.

‘unknown^b^’ represents genes without Gene Ontology annotation.

**Figure 2:**
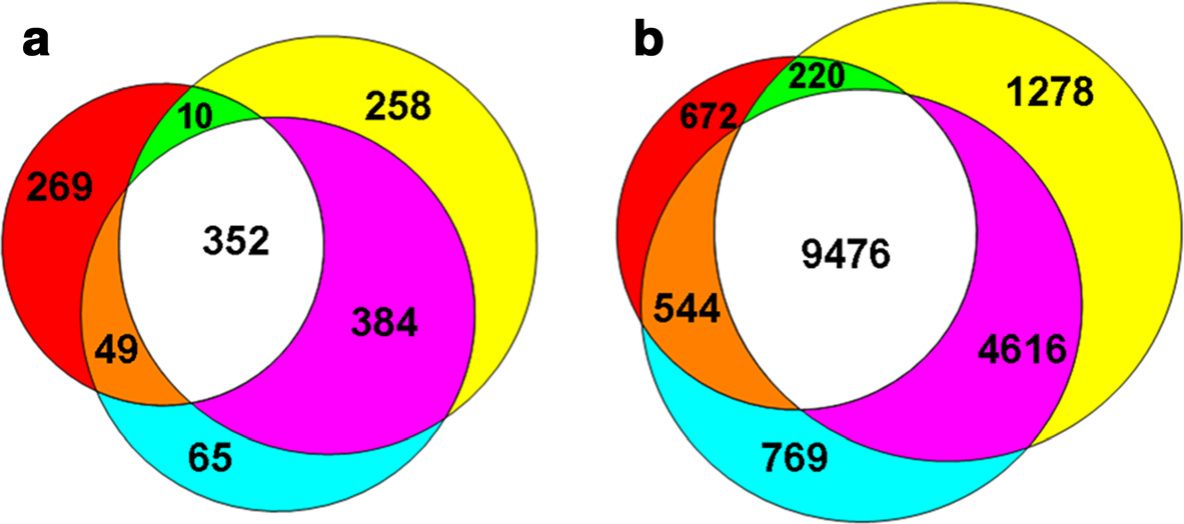
** Venn diagram showing overlaps among up**-**regulated genes during soybean seed development.** (**a**). All the unigenes that showed increases of at least 2-fold (log_2_ratio ≥ 1) in the 3 contrasts, (**b**). All the unigenes that showed decreases of at least 2-fold (log_2_ratio ≤ 1) in the 3 contrasts. Number in one circle denotes stage-specific genes, and number in two or more intersecting circles denotes overlapped genes.

There were 680, 1004, and 850 up-regulated unigenes and 10912, 15590, and 15405 down-regulated unigenes in the three respective groups (Table 1). Among the up-regulated unigenes, 352 were co-expressed genes showing increases of at least 2-fold (log_2_ratio ≥ 1), and 269, 258, and 65 of the up-regulated genes were specific to each of the three respective groups. Among the down-regulated unigenes, 9476 were co-expressed genes showing decreases of at least 2-fold (log_2_ratio ≤ 1), and 672, 1278, and 769 of the down-regulated genes were specific to each of the three respective groups (Figure 2).

Differentially expressed genes provide clues about the molecular events related to seed development. Further investigation of highly expressed genes may be warranted to determine what functional roles the highly expressed sequences may play in soybean seeds. To understand the relationship between the time at which unigenes are co-expressed and their biological significance, we carried out Gene Ontology enrichment analysis.

### Gene ontology category analysis and DEGs in each category

**Figure 3:**
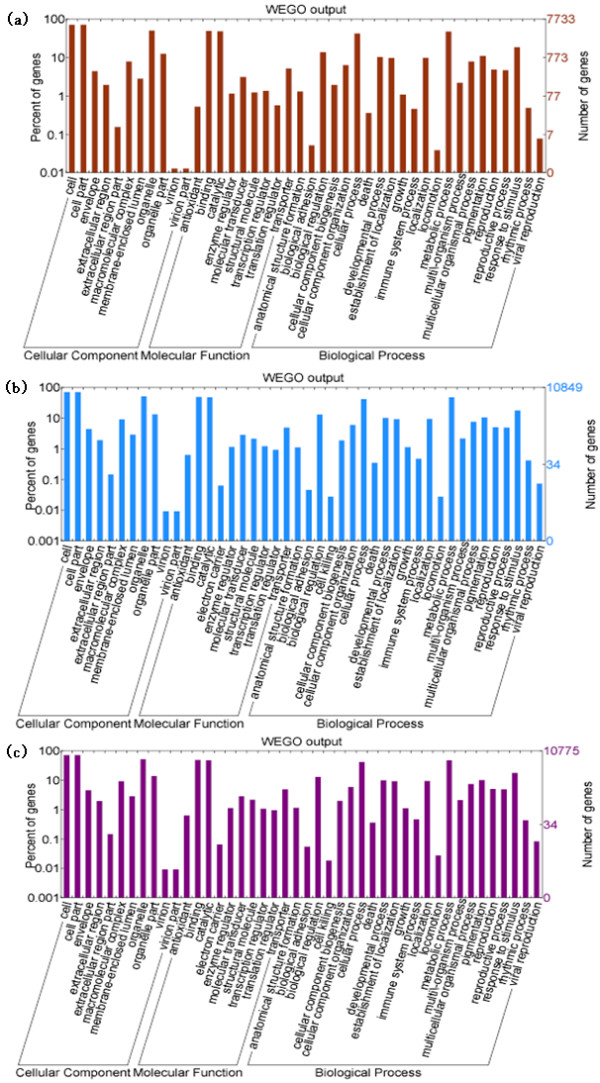
** Functional categorization of significantly differentially expressed genes during seed development.** Functional categorization was performed using BGI WEGO (Web Gene Ontology Annotation Plot). (**a**). The functional categorization of differentially expressed genes between 35 DAF and 15 DAF. (**b**). The functional categorization of differentially expressed genes between 55 DAF and 15 DAF. (**c**). The functional categorization of differentially expressed genes between 65 DAF and 15 DAF.

As a useful tool for gene functional annotation, WEGO (Web Gene Ontology Annotation Plot) has been widely used in many important studies, including the rice genome project and the silkworm genome project [12-14]. It has become one of the most commonly used tools for downstream gene annotation analysis, especially in comparative genomics studies. In this research, WEGO analysis assigned the DEGs (FDR ≤ 0.001 and |log_2_ratio| ≥ 1) to three functional categories; Cellular Component, Molecular Function, or Biological Process. A total of 11963 unigenes had at least one GO functional category. In the three contrast groups, all differentially expressed genes with a Gene Ontology annotation were further classified into subsets. There were 11 subsets within the Cellular Component category, 10 subsets within the Molecular Function category, and 23 subsets within the Biological Process category (Figure 3). Thus, the most abundant unigenes were related to binding and catalytic activity in the Molecular Function category, cell, cell parts and organelles in the Cellular Component category, and cellular and metabolic in the Biological Process category. The results showed that these types of genes were highly enriched in our soybean transcriptomes. In terms of the categories, the three contrast groups showed similar patterns. 

Because we are interested in lipid biosynthesis, we focused on genes associated with this process that were differentially expressed during seed development. From the Component Ontology data, eight differentially co-expressed lipid particle unigenes were identified. Among them, seven unigenes were up-regulated, and one unigene was down-regulated during seed development. From the Function Ontology data, unigenes associated with lipid biosynthesis could be further classified into three categories: those with lipid kinase activity (9 differentially co-expressed down-regulated unigenes); those with fatty acid ligase activity (6 differentially co-expressed down-regulated unigenes); and those with fatty acid synthase activity (18 differentially co-expressed unigenes, 4 of which were up-regulated and 14 down-regulated). From the Process Ontology data, unigenes associated with lipid biosynthesis were in two categories; lipid biosynthetic process (78 differentially co-expressed unigenes, 5 of which were up-regulated, and 73 down-regulated), and fatty acid biosynthetic process (49 differentially co-expressed unigenes, 4 of which were up-regulated, and 45 down-regulated). Interesting, the 49 co-expressed unigenes detected in the fatty acid biosynthetic process were all included in unigenes detected in the lipid biosynthetic process. Among the 113 unigenes analyzed by Gene Ontology, 15 co-expressed unigenes that showed high levels of expression (log_2_fold values ≥ 1) could be responsible for lipid biosynthesis (Table 2).

**Table 2:** DEGs identity, expression ratios, and Gene Ontology annotations

**Unigene ID**	**Gene ID**	**Accession No**	**Annotation**	**log**_**2**_**ratio**		
				**35 DAF**	**55 DAF**	**65 DAF**
**gnl|UG|Gma#S15927547**	**31463931**	**CD405959**	**Catalytic activity, mRNA sequence**	**−12.45**	**−12.45**	**−12.45**
**gnl|UG|Gma#S15936780**	**31473219**	**CD415247**	**Gm_ck5311 Soybean induced by salicylic acid*G. max*cDNA 3'**	**−1.52**	**−5.95**	**−13.86**
**gnl|UG|Gma#S21537460**	**51335477**	**CO979343**	**Nucleoside-triphosphatase activity**	**−1.69**	**−2.00**	**−1.27**
**gnl|UG|Gma#S21568089**	**51344862**	**CO985595**	**Intracellular membrane-bounded organelle**	**−1.85**	**−14.73**	**−4.59**
**gnl|UG|Gma#S21568290**	**51344703**	**CO985436**	**GM89024B1A03.r1 Gm-r1089*G. max*cDNA**	**−2.16**	**−2.20**	**−3.15**
**gnl|UG|Gma#S23063462**	**58016474**	**CX703216**	**Water stressed 48 h segment 2 gmrtDrNS01*G. max*cDNA 3'**	**−1.81**	**−1.39**	**−1.76**
**gnl|UG|Gma#S23071366**	**58024339**	**CX711080**	**Oxidoreductase activity**	**−8.01**	**−11.12**	**−11.05**
**gnl|UG|Gma#S39303981**	**151397976**	**EV267849**	**Carbon-carbon lyase activity**	**−6.22**	**−5.33**	**−13.86**
**gnl|UG|Gma#S39304525**	**151398522**	**EV268393**	**GLMA586TF JCVI-SOY2*G. max*cDNA 5'**	**−4.60**	**−2.39**	**−2.32**
**gnl|UG|Gma#S39310377**	**151405779**	**EV274245**	**GLMC444TF JCVI-SOY2*G. max*cDNA 5'**	**−2.62**	**−1.68**	**−2.04**
**gnl|UG|Gma#S39548296**	**152926331**	**EU003576**	**Oxidoreductase activity**	**−6.08**	**−10.80**	**−10.22**
**gnl|UG|Gma#S45535109**	**192301009**	**FG990109**	**GLLD114TF JCVI-SOY1*G. max*cDNA 5'**	**−1.60**	**−1.97**	**−2.13**
**gnl|UG|Gma#S45548971**	**192312746**	**FK003972**	**GLMDD92TR JCVI-SOY2*G. max*cDNA 5'**	**−1.23**	**−1.07**	**−1.57**
**gnl|UG|Gma#S45553331**	**192318756**	**FK008332**	**Oxidoreductase activity**	**−4.38**	**−2.62**	**−6.01**
**gnl|UG|Gma#S46507969**	**193399415**	**FK361305**	**Soybean Seeds Containing Globular-Stage Embryos*G. max*cDNA, mRNA sequence**	**−2.95**	**−4.39**	**−14.46**
**gnl|UG|Gma#S46512605**	**193402683**	**FK365941**	**Oxidoreductase activity**	**−3.98**	**−4.68**	**−5.61**
**gnl|UG|Gma#S47453769**	**208298984**	**GE091511**	**Oxidoreductase activity, mRNA sequence**	**−3.28**	**−14.04**	**−14.04**
**gnl|UG|Gma#S47698257**	**209701642**	**BW673128**	**Oxidoreductase activity, mRNA sequence**	**−3.89**	**−13.83**	**−4.34**
**gnl|UG|Gma#S48312113**	**210145949**	**AK244640**	**Oxidoreductase activity**	**−2.54**	**−4.24**	**−2.95**
**gnl|UG|Gma#S48312226**	**210145836**	**AK244527**	**Oxidoreductase activity**	**−1.22**	**−2.76**	**−2.62**
**gnl|UG|Gma#S48312292**	**210145770**	**AK244461**	***G. max*cDNA**	**−5.08**	**−14.02**	**−5.71**
**gnl|UG|Gma#S48312360**	**210145702**	**AK244393**	***G. max*cDNA**	**−7.45**	**−10.67**	**−9.92**
**gnl|UG|Gma#S48312476**	**210145586**	**AK244277**	***G. max*cDNA**	**−2.29**	**−2.99**	**−2.62**
**gnl|UG|Gma#S48312873**	**210145189**	**AK243880**	***G. max*cDNA**	**−5.00**	**−7.34**	**−7.85**
**gnl|UG|Gma#S48313629**	**210144071**	**AK286853**	**Oxidoreductase activity**	**−5.01**	**−6.81**	**−6.93**
**gnl|UG|Gma#S48313665**	**210144035**	**AK286817**	**Oxidoreductase activity**	**−1.60**	**−2.61**	**−2.20**
**gnl|UG|Gma#S48314836**	**210142864**	**AK285646**	**Oxidoreductase activity**	**−5.68**	**−10.31**	**−10.25**
**gnl|UG|Gma#S48315365**	**210142183**	**AK246102**	***G. max*cDNA**	**−12.13**	**−2.15**	**−2.09**
**gnl|UG|Gma#S48315795**	**210141753**	**AK245672**	**Threonine kinase activity**	**−1.11**	**−1.38**	**−1.25**
**gnl|UG|Gma#S48315845**	**210141703**	**AK245622**	**Fatty acid ligase activity**	**−4.52**	**−5.95**	**−5.30**
**gnl|UG|Gma#S48316028**	**210141520**	**AK245439**	**Fatty acid synthase activity**	**−2.84**	**−6.12**	**−6.05**
**gnl|UG|Gma#S48316351**	**210141197**	**AK245114**	**Fatty acid synthase activity**	**−2.05**	**−11.17**	**−2.90**
**gnl|UG|Gma#S48316398**	**210141150**	**AK245067**	***G. max*cDNA**	**−2.16**	**−3.24**	**−3.39**
**gnl|UG|Gma#S48316494**	**210141054**	**AK244973**	**Oxidoreductase activity**	**−4.06**	**−3.80**	**−3.91**
**gnl|UG|Gma#S48316776**	**210140772**	**AK244691**	***G. max*cDNA**	**−1.54**	**−2.50**	**−2.62**
**gnl|UG|Gma#S48540273**	**213602571**	**DB978070**	**Transferase activity, mRNA sequence**	**−11.98**	**−11.98**	**−11.98**
**gnl|UG|Gma#S4876535**	**6070118**	**AW099690**	**Oxidoreductase activity**	**−12.38**	**−12.38**	**−12.38**
**gnl|UG|Gma#S4928292**	**7924555**	**AW830581**	***G. max*cDNA clone**	**−1.09**	**−1.79**	**−1.06**
**gnl|UG|Gma#S4989750**	**9983486**	**BE657594**	**oxidoreductase activity, mRNA sequence**	**−2.95**	**−12.36**	**−12.36**
**gnl|UG|Gma#S4991939**	**10252627**	**BE820393**	**fatty acid ligase activity**	**−1.44**	**−3.87**	**−3.22**
**gnl|UG|Gma#S5146402**	**505137**	**D13949**	***G. max*lipoxygenase-2 mRNA**	**8.72**	**7.41**	**8.23**
**gnl|UG|Gma#S5146516**	**2739009**	**AF022464**	***G. max*cytochrome P450 monooxygenase**	**−2.15**	**−3.03**	**−3.11**
**gnl|UG|Gma#S5146617**	**2270993**	**AF004809**	***G. max*Ca + 2-bingding EF hand protein**	**2.75**	**10.33**	**9.51**
**gnl|UG|Gma#S5146661**	**18674**	**X67304**	***G. max*mRNA for lipoxygenase-1**	**10.25**	**9.44**	**9.91**
**gnl|UG|Gma#S5146662**	**1794171**	**U50081**	***G. max*lipoxygenase-3 mRNA**	**9.25**	**8.86**	**9.23**
**gnl|UG|Gma#S5146890**	**43985**	**U04785**	***G. max*Williams 82 lipoxygenase mRNA**	**−5.46**	**−8.74**	**−8.67**
**gnl|UG|Gma#S53086049**	**255645378**	**BT097946**	**Unknown mRNA**	**−2.10**	**−4.42**	**−4.06**
**gnl|UG|Gma#S53088884**	**255638127**	**BT095104**	**Unknown mRNA**	**−1.83**	**−2.30**	**−3.82**
**gnl|UG|Gma#S53090173**	**255635620**	**BT093810**	**fatty acid synthase activity, unknown mRNA**	**−1.52**	**−3.36**	**−2.78**
**gnl|UG|Gma#S52654816**	**254332339**	**GR846384**	**Monooxygenase activity**	**−2.25**	**−2.78**	**−3.29**
**gnl|UG|Gma#S39313094**	**151407147**	**EV276962**	**GLMCZ08TF JCVI-SOY2,*G. max*cDNA 5' mRNA sequence**	**−1.16**	**−2.35**	**−2.54**
**gnl|UG|Gma#S53090215**	**255635538**	**BT093768**	**Oxidoreductase activity,unknown mRNA**	**−1.39**	**−1.64**	**−2.06**
**gnl|UG|Gma#S4993394**	**10254082**	**BE821848**	**Fatty acid synthase activity, mRNA sequence**	**−1.21**	**−2.19**	**−1.78**
**gnl|UG|Gma#S48316085**	**210141463**	**AK245382**	**UDP-glycosyltransferase activity**	**−1.63**	**−2.06**	**−1.84**
**gnl|UG|Gma#S6669271**	**26046073**	**CA783412**	**Oxidoreductase activity, mRNA sequence**	**−3.45**	**−6.81**	**−4.05**
**gnl|UG|Gma#S48315123**	**210142573**	**AK285451**	**Catalytic activity**	**−1.51**	**−6.89**	**−4.90**
**gnl|UG|Gma#S48313908**	**210143792**	**AK286574**	***G. max*cDNA**	**−2.07**	**−3.73**	**−3.34**
**gnl|UG|Gma#S48316204**	**210141408**	**AK245263**	**Oxidoreductase activity**	**−1.32**	**−2.34**	**−2.35**
**gnl|UG|Gma#S39303626**	**151397621**	**EV267494**	**Oxidoreductase activity**	**−1.05**	**−1.32**	**−1.06**
**gnl|UG|Gma#S48316577**	**210140971**	**AK244890**	**Methyltransferase activity**	**−1.22**	**−3.23**	**−2.49**
**gnl|UG|Gma#S32218187**	**90658387**	**DQ394572**	***G. max*cytochrome P450 monooxygenase CYP97C10 (CYP97C10) mRNA**	**−3.05**	**−1.25**	**−2.12**
**gnl|UG|Gma#S48314962**	**210142734**	**AK285227**	**Cyclase activity**	**−2.25**	**−3.28**	**−2.84**
**gnl|UG|Gma#S53089425**	**255637080**	**BT094559**	**Unknown mRNA**	**−2.16**	**−4.59**	**−5.52**
**gnl|UG|Gma#S52634774**	**254317397**	**GR826342**	**Oxidoreductase activity**	**−1.60**	**−2.39**	**−3.02**
**gnl|UG|Gma#S21537312**	**51335213**	**CO979079**	**mRNA sequence, mRNA sequence**	**−3.48**	**−5.92**	**−4.96**
**gnl|UG|Gma#S48316562**	**210140986**	**AK244905**	***G. max*cDNA**	**−2.12**	**−1.57**	**−1.29**
**gnl|UG|Gma#S23065186**	**58018200**	**CX704942**	**Monooxygenase activity**	**−5.00**	**−12.44**	**−12.44**
**gnl|UG|Gma#S5146686**	**1399379**	**U43683**	**C-methyltransferase activity**	**−1.65**	**−2.28**	**−2.35**
**gnl|UG|Gma#S53085203**	**255647037**	**BT098796**	**C-4 methylsterol oxidase activity, unknown mRNA**	**−1.24**	**−2.26**	**−1.68**
**gnl|UG|Gma#S53086564**	**255644365**	**BT097429**	**Unknown mRNA**	**−1.45**	**−2.73**	**−3.21**
**gnl|UG|Gma#S48313496**	**210144204**	**AK286986**	***G. max*cDNA**	**−1.70**	**−3.40**	**−3.21**
**gnl|UG|Gma#S53089772**	**255636400**	**BT094211**	**Geranyltranstransferase activity, unknown mRNA**	**−1.62**	**−1.73**	**−2.25**
**gnl|UG|Gma#S39313896**	**151407949**	**EV277764**	**UDP-galactosyltransferase activity**	**−3.83**	**−5.75**	**−14.29**
**gnl|UG|Gma#S52663531**	**254341371**	**GR855099**	**Small conjugating protein ligase activity**	**−1.65**	**−1.50**	**−2.21**
**gnl|UG|Gma#S53088132**	**255639591**	**BT095858**	**Unknown mRNA**	**1.19**	**−1.17**	**−1.02**
**gnl|UG|Gma#S53085581**	**255646289**	**BT098415**	**3-hydroxyacyl-[acyl-carrier-protein] dehydratase activity unknown mRNA**	**−2.63**	**−5.89**	**−5.38**
**gnl|UG|Gma#S30676923**	**85001710**	**DQ340246**	***G. max*cytochrome P450 monooxygenase CYP90A15 (CYP90A15) mRNA**	**−2.18**	**−4.17**	**−3.39**
**gnl|UG|Gma#S5142779**	**22931631**	**BU548770**	**Gm-r1088*G. max*cDNA clone Gm-r1088-6501 3'**	**−4.74**	**−7.44**	**−7.37**
**gnl|UG|Gma#S39312371**	**151406424**	**EV276239**	**Phosphatidylinositol phosphate kinase activity**	**−4.60**	**−13.22**	**−13.22**
**gnl|UG|Gma#S22952836**	**57576101**	**CX549072**	**Lipid kinase activity**	**−1.46**	**−2.12**	**−2.30**
**gnl|UG|Gma#S46837027**	**193692332**	**FK631119**	**Lipid kinase activity**	**−2.54**	**−2.17**	**−4.90**
**gnl|UG|Gma#S23071197**	**58024263**	**CX711004**	**Lipid kinase activity**	**−1.34**	**−1.55**	**−1.81**
**gnl|UG|Gma#S21568721**	**51345091**	**CO985824**	**Phosphatidylinositol phosphate kinase activity**	**−2.00**	**−2.25**	**−2.70**
**gnl|UG|Gma#S47246628**	**208083682**	**GD884374**	**Soybean Seeds Containing Globular-Stage Embryos*G. max*cDNA mRNA sequence**	**−2.65**	**−3.77**	**−2.38**
**gnl|UG|Gma#S23061755**	**58014763**	**CX701505**	**Phosphatidylinositol phosphate kinase activity**	**−2.37**	**−4.07**	**−2.51**
**gnl|UG|Gma#S48551001**	**214004471**	**DB984819**	**Lipid kinase activity, mRNA sequence**	**−1.83**	**−3.59**	**−3.28**
**gnl|UG|Gma#S18957041**	**42723029**	**CK768928**	**Phosphoinositide 3-kinase activity**	**−1.27**	**−1.40**	**−1.05**
**gnl|UG|Gma#S4989748**	**9983484**	**BE657592**	**CoA-ligase activity**	**−5.06**	**−16.02**	**−16.02**
**gnl|UG|Gma#S39311670**	**151405724**	**EV275538**	**Fatty acid ligase activity, mRNA sequence**	**−3.25**	**−2.95**	**−2.88**
**gnl|UG|Gma#S45544712**	**192308617**	**FG999712**	**Fatty acid ligase activity**	**−2.12**	**−12.47**	**−4.75**
**gnl|UG|Gma#S4994916**	**10255604**	**BE823370**	**Gm-r1070*G. max*cDNA clone Gm-r1070-7609 3'mRNA sequence**	**−1.10**	**−14.60**	**−4.11**
**gnl|UG|Gma#S53085813**	**255645838**	**BT098182**	**Unknown mRNA**	**8.39**	**10.22**	**9.95**
**gnl|UG|Gma#S22952816**	**57576081**	**CX549052**	**Fatty acid synthase activity**	**−1.25**	**−1.67**	**−2.11**
**gnl|UG|Gma#S48312148**	**210145914**	**AK244605**	**Fatty acid synthase activity**	**−1.30**	**−3.09**	**−3.12**
**gnl|UG|Gma#S53090057**	**255635847**	**BT093926**	**Unknown mRNA, fatty acid synthase activity**	**6.68**	**7.34**	**7.40**
**gnl|UG|Gma#S18957150**	**42723162**	**CK769061**	**Fatty acid synthase activity, mRNA sequence**	**4.08**	**6.59**	**6.11**
**gnl|UG|Gma#S48312652**	**210145410**	**AK244101**	**Acyl-[acyl-carrier-protein] hydrolase activity**	**−1.39**	**−2.41**	**−2.69**
**gnl|UG|Gma#S5129103**	**16345261**	**BI970856**	**Fatty acid synthase activity**	**−1.51**	**−4.84**	**−4.01**
**gnl|UG|Gma#S48315227**	**210142469**	**AK285347**	**Fatty acid synthase activity**	**−1.24**	**−3.64**	**−3.25**
**gnl|UG|Gma#S53090055**	**255635851**	**BT093928**	**Fatty acid synthase activity unknown mRNA**	**−2.14**	**−1.37**	**−2.12**
**gnl|UG|Gma#S5146394**	**7862173**	**AF260565**	***G. max*beta-ketoacyl-acyl carrier protein synthase III mRNA**	**−1.10**	**−2.05**	**−2.09**
**gnl|UG|Gma#S5146406**	**7385202**	**AF243183**	***G. max*beta-ketoacyl-ACP synthetase I-2 mRNA**	**−1.64**	**−3.36**	**−3.78**
**gnl|UG|Gma#S23063275**	**58016255**	**CX702997**	**Fatty acid synthase activity**	**−1.48**	**−1.89**	**−2.94**
**gnl|UG|Gma#S48316073**	**210141475**	**AK245394**	***G. max*cDNA**	**6.81**	**7.52**	**7.48**
**gnl|UG|Gma#S4934087**	**6846492**	**AW348782**	**Fatty acid synthase activity, mRNA sequence**	**−3.49**	**−5.72**	**−4.91**
**gnl|UG|Gma#S45541550**	**192306090**	**FG996550**	**Lipid particle**	**4.21**	**3.89**	**3.84**
**gnl|UG|Gma#S48531444**	**213596804**	**DB978036**	**Lipid particle**	**4.87**	**5.13**	**4.79**
**gnl|UG|Gma#S48316140**	**210141408**	**AK245327**	**Lipid particle**	**−1.32**	**−2.17**	**−2.61**
**gnl|UG|Gma#S18957317**	**42723121**	**CK769020**	**Lipid particle (GmPM13) mRNA**	**3.75**	**3.86**	**4.05**
**gnl|UG|Gma#S48312213**	**210145849**	**AK244540**	**Lipid particle**	**5.29**	**5.12**	**5.03**
**gnl|UG|Gma#S18956788**	**42722719**	**CK768618**	**Lipid particle**	**4.63**	**4.51**	**4.48**
**gnl|UG|Gma#S4995024**	**10255768**	**BE823478**	***G. max*mRNA sequence**	**8.05**	**8.80**	**8.79**
**gnl|UG|Gma#S4899715**	**6747398**	**AW317854**	**Lipid particle**	**4.64**	**4.75**	**4.74**

### Annotation of lipid-relevant genes in fatty acid pathway

**Figure 4:**
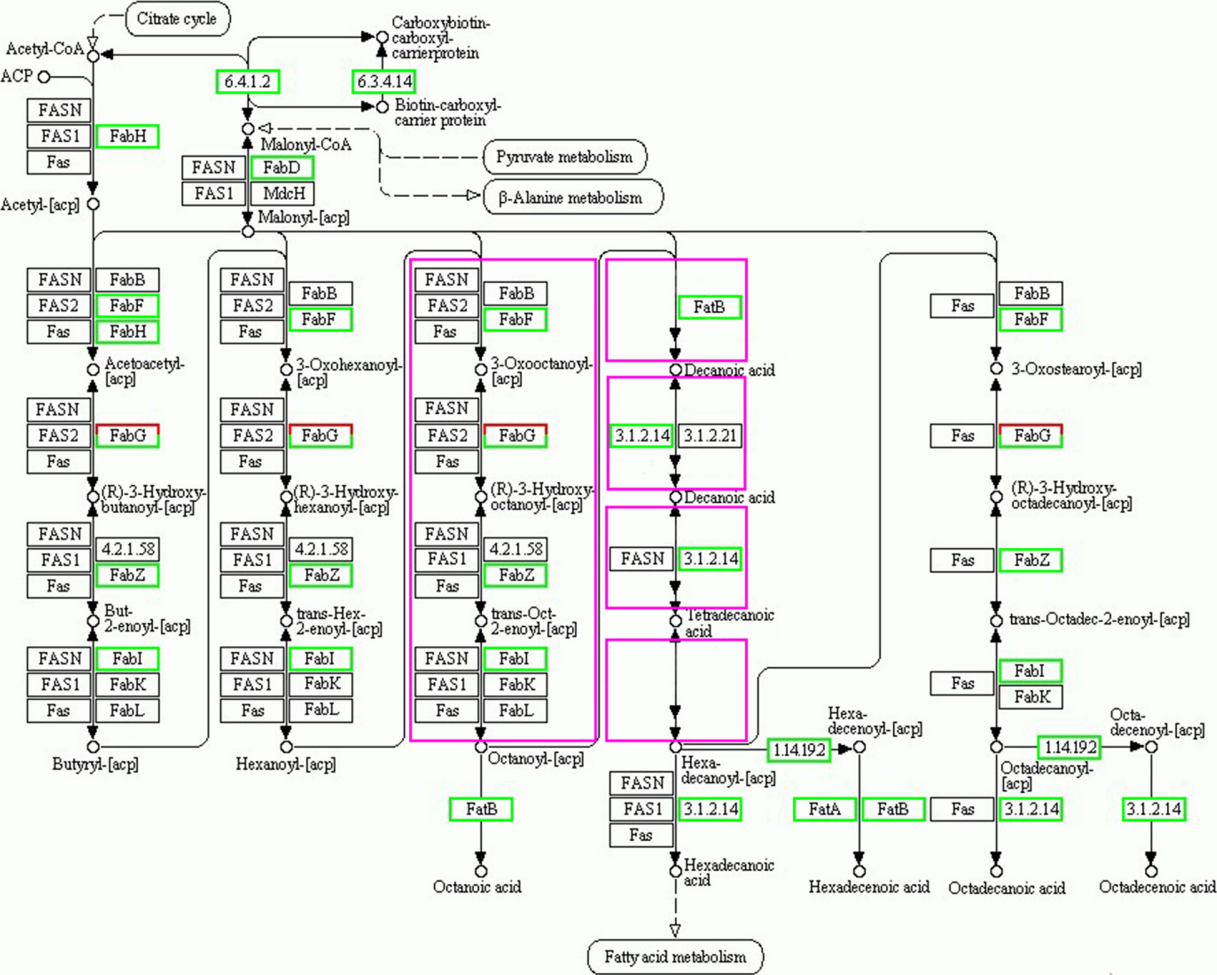
** Fatty acid pathway in soybean.** Processes in pink rectangles are shown in abbreviated form. Red or green rectangles indicate up-regulated or down-regulated genes, respectively. *FAS*N, Fas: fatty acid synthase; *FAS*1, 2: fatty acid synthase subunit beta, alpha; *Fab*A: 3-hydroxydecanoyl-[acyl-carrier-protein] dehydratase; *Fab*B, *Fab*F, *Fab*H: 3-oxoacyl-[acyl-carrier-protein] synthase I, II, III; *Fab*D: [acyl-carrier-protein] S-malonyltransferase; *Fab*G: 3-oxoacyl-[acyl-carrier protein] reductase; *Fab*I, *Fab*K, *Fab*L: enoyl-[acyl-carrier protein] reductase I, II, III; *Fab*Z: 3R-hydroxymyristoyl ACP dehydrase; MdcH: malonate decarboxylase epsilon subunit; *Fat*A, *Fat*B: fatty acyl-ACP thioesterase A, B.

Genes usually interact with each other to carry out certain biological functions. Knowledge of the expressions of multiple genes and their regulation during oil biosynthesis is required to further understand the regulatory mechanisms controlling oil metabolism. Pathway-based analysis helps to clarify the biological functions of genes, and identifies significantly enriched metabolic pathways or signal transduction pathways associated with the DEGs compared with the whole genome background. For our research, 124 biological pathways, including the starch and sucrose metabolism pathway, the citrate cycle pathway, the fatty acid biosynthesis pathway, and many others were identified by KEGG pathway analysis of unigenes. A total of 4836, 6796, and 6758 DEGs with pathway annotations were identified in the three respective contrast groups. From those pathways, we selected the fatty acid biosynthesis pathway (Figure 4) for deep analysis. In the three contrast groups, 24 DEGs associated with the fatty acid pathway were co-expressed (Table 3). Interestingly, 13 of the identified unigenes were the same as those identified from the GO analysis. Among those 24 DGEs, only four ungenes (BT098182, AK245394, BT093926, BT093412) were up-regulated, which are encoding 3-oxoacyl-[acyl-carrier protein] reductases (*Fab*G) and were annotated as having fatty acid synthase activity. The other 20 unigenes were down-regulated, which may be the negatively controlled genes in the fatty acid biosynthesis pathway. Figure 4 shows the locations of the differentially co-expressed unigenes in the fatty acid pathway. Those shown in red and green represent genes that were up-regulated and down-regulated, respectively, during seed development.

**Table 3:** DEGs involved in fatty acid pathway

**Unigene ID**	**Gene ID**	**Accession No**	**Annotation**	**log**_2_**ratio**		
				**35 DAF**	**55 DAF**	**65 DAF**
**gnl|UG|Gma#S53085813**	**255645838**	**BT098182**	**Unknown mRNA**	**8.39**	**10.22**	**9.95**
**gnl|UG|Gma#S48316073**	**210141475**	**AK245394**	**G. max cDNA**	**6.81**	**7.52**	**7.48**
**gnl|UG|Gma#S53090057**	**255635847**	**BT093926**	**Unknown mRNA, fatty acid synthase activity**	**6.68**	**7.34**	**7.40**
**gnl|UG|Gma#S53090570**	**255634845**	**BT093412**	**Catalytic activity, unknown mRNA**	**3.88**	**5.09**	**4.29**
**gnl|UG|Gma#S53086639**	**255642528**	**BT097354**	**Oxidoreductase activity, unknown mRNA**	**−10.89**	**−10.89**	**−10.89**
**nl|UG|Gma#S53085581**	**255646289**	**BT098415**	**3-Hydroxyacyl-[acyl-carrier-protein] dehydratase activity, unknown mRNA**	**−2.63**	**−5.89**	**−5.38**
**gnl|UG|Gma#S4934087**	**6846492**	**AW348782**	**Fatty acid synthase activity, mRNA sequence**	**−3.49**	**−5.72**	**−4.91**
**gnl|UG|Gma#S53089253**	**255637417**	**BT094733**	**Unknown mRNA**	**−2.01**	**−4.98**	**−4.68**
**gnl|UG|Gma#S5129103**	**16345261**	**BI970856**	**Fatty acid synthase activity**	**−1.51**	**−4.84**	**−4.01**
**gnl|UG|Gma#S48312866**	**210145196**	**AK243887**	**Metabolic process**	**−2.25**	**−4.42**	**−3.98**
**gnl|UG|Gma#S5146406**	**7385202**	**AF243183**	***G. max*beta-ketoacyl-ACP synthetase I-2 mRNA**	**−1.64**	**−3.36**	**−3.78**
**gnl|UG|Gma#S5146689**	**1143321**	**U40979**	***G. max*alfa-carboxyltransferase (accA-2) precursor mRNA**	**−1.19**	**−5.05**	**−3.66**
**gnl|UG|Gma#S53087052**	**255641716**	**BT096940**	**Catalytic activity, unknown mRNA**	**−1.63**	**−2.74**	**−3.27**
**gnl|UG|Gma#S48315227**	**210142469**	**AK285347**	**Fatty acid synthase activity**	**−1.24**	**−3.64**	**−3.25**
**gnl|UG|Gma#S48312148**	**210145914**	**AK244605**	**Fatty acid synthase activity**	**−1.30**	**−3.09**	**−3.12**
**gnl|UG|Gma#S23063275**	**58016255**	**CX702997**	**Fatty acid synthase activity**	**−1.48**	**−1.89**	**−2.94**
**gnl|UG|Gma#S5146375**	**9621819**	**AF165159**	***G. max*carboxyl transferase alpha subunit (accA-3) mRNA**	**−1.16**	**−4.06**	**−2.86**
**gnl|UG|Gma#S48312652**	**210145410**	**AK244101**	**Acyl-[acyl-carrier-protein] hydrolase activity**	**−1.39**	**−2.41**	**−2.69**
**gnl|UG|Gma#S53089095**	**255637722**	**BT094891**	**Unknown mRNA**	**−1.05**	**−2.54**	**−2.53**
**gnl|UG|Gma#S48316546**	**210141002**	**AK244921**	**Oxidoreductase activity**	**−1.56**	**−1.99**	**−2.43**
**gnl|UG|Gma#S48316068**	**210141480**	**AK245399**	**CoA carboxylase activity**	**−1.28**	**−2.57**	**−2.17**
**gnl|UG|Gma#S22952816**	**57576081**	**CX549052**	**Fatty acid synthase activity**	**−1.25**	**−1.67**	**−2.11**
**gnl|UG|Gma#S5146394**	**7862173**	**AF260565**	***G. max*beta-ketoacyl-acyl carrier protein synthase III mRNA**	**−1.10**	**−2.05**	**−2.09**
**gnl|UG|Gma#S23063462**	**58016474**	**CX703216**	**Water-stressed 48 h segment 2 gmrtDrNS01*G. max*cDNA 3'**	**−1.81**	**−1.39**	**−1.76**

### Clustering of candidate genes

**Figure 5:**
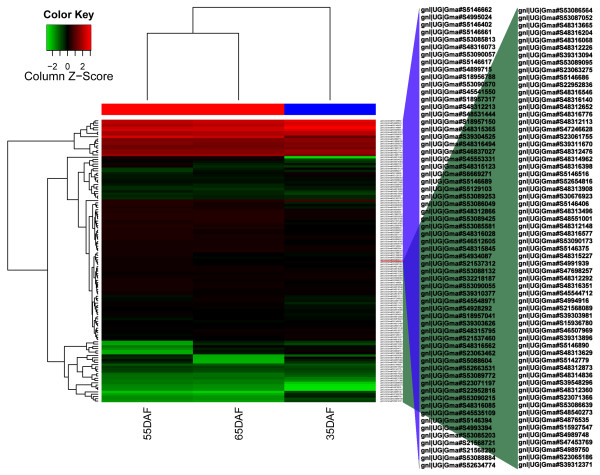
** Expression changes and cluster analysis of genes (124 in total) that were differentially expressed between 35 DAF and 15 DAF seeds (control), 55 DAF and 15 DAF seeds, and 65 DAF and 15 DAF seeds.** Cluster analysis of genes was performed using R program. Rows represent differentially expressed genes, columns represent different contrast groups. Red, green, and black boxes represent genes showing increased, decreased, or equal expression levels, respectively. Color saturation reflects magnitude of log_2_ expression ratio for each gene.

A total of 124 unigenes, including those detected by GO and pathway analyses, were selected for clustering analysis. All of these unigenes showed different expression profiles. The genes were clustered according to the similarity of their expression patterns. A subset of the data is shown in Figure 5. The heatmap of the RPKM normalized log_2_-transformed transcription count was generated using R programs. Up-regulated unigenes are shown in red, while down regulated ones are shown in green. This analysis allowed us to define common qualitative patterns in gene expression changes over time. Our results suggested that only a few genes were expressed differentially in the three contrast groups, and that the majority of the transcriptome had approximately similar expression levels. In this manner, similar patterns were clustered together in the same manner as a taxonomic tree. Therefore, we can speculate on the roles of unigenes of unknown function by comparison to the annotated genes with known functions in the same cluster.

### Confirmation of differentially expressed genes by qRT-PCR

To confirm the expression patterns determined by Solexa RNA-sequencing analysis, we used qRT-PCR analyses to analyze expressions of 12 candidate genes (Additional file 4: Table S4). The soybean tubulin gene was used as an internal control. Although the Solexa log_2_-fold values showed slight variations compared with the corresponding values from the qRT-PCR analyses, the expression data from Solexa RNA-seq analysis was largely consistent with those obtained by qRT-PCR (Additional file 6: Figure S2).

## Discussion

Lipid synthesis depends on the correct spatial and temporal activity of many gene products. These genes execute their function in three stages: fatty acid synthesis in the plastid, triacylglycerol (TAG) synthesis in the endoplasmic reticulum (ER), and assembly into an oil body. Significant improvements in oil accumulation must be accompanied by changes in activity of the genes involved in fatty acid biosynthesis in developing seeds. Identification of these genes and their regulatory pathway would provide not only new genetic information for understanding soybean seed development, but also for controlling gene expression in developing seeds to alter oil accumulation.

This study has provided a new data set identifying the expression of DEGs during oil accumulation in developing seeds. Massive parallel sequencing identified 11592, 16594 and 16255 differentially expressed unigenes from three contrasting libraries covering four stages of seed development. We examined gene expression levels in detail, and found significant differences among the four growth stages. Among the differentially expressed genes, more were down-regulated than up-regulated, and some showed a differential expression pattern in all three contrast groups, indicating that there were overlaps at the transcriptional level. The fact that there were many down-regulated genes indicates that there are more negatively regulated genes than positively regulated ones with functions in the fatty acid pathway. However, it does not mean that lower expression of these genes leads to lower oil contents, because of the complexity of the lipid biosynthetic pathway. To identify the genes associated with lipid biosynthesis, we determined gene expression patterns at specific stages of seed development, and conducted a GO analysis. To explore the genes with unknown functions, the expression patterns of 124 unigenes were analyzed by hierarchical clustering according to similarities in expression profiles across all conditions.

In the lipid biosynthetic pathway, acetyl CoA carboxylase (ACCase) plays an important regulatory role. ACCase catalyzes the carboxylation of acetyl-CoA to malonyl-CoA [15], which is then transacylated by malonyl-CoA-acyl carrier protein transacylase (*Fab*D or *MCAT,*[EC 2.3.1.39]) to the acyl carrier protein (ACP), forming malonyl ACP. The latter adds a 2-carbon acetyl unit to the nascent or growing fatty acyl chain. The basic reaction of fatty acid synthesis is to combine molecules composed of two carbon units into longer chains to form fatty acids. The initial condensation of 2-carbon units is catalyzed by β-ketoacyl-ACP synthase III (*KAS*III, EC2.3.1.180) which uses acetyl-CoA and malonyl-ACP as substrates. After the two reductions and dehydration reactions, a 4-carbon fatty acid, butyrate, is produced. This is poorly condensed by *KAS*III but is a good substrate for *KAS*I, which elongates 4-carbon chains to 14-carbon chains.

In this study, we studied in detail the genes with key roles in lipid biosynthesis. Very frequently, the same enzymatic function is redundantly encoded by several unigenes. This may be the result of different proteins referenced with the same EC number or it may represent different transcripts encoding specific enzyme subunits. This situation was significant in the present study. Most plants have two forms of ACCase, the homomeric form in the cytosol, composed of a single large polypeptide catalyzing the individual carboxylation reactions [16], and the heteromeric form in plastids, composed of four subunits; biotin carboxylase (BC) [17], biotin carboxyl carrier protein (BCCP) [18], α-carboxyltransferase (α-CT) [19] and β-carboxyltransferase (β-CT) [20]. In the present study, seven co-expressed unigenes (GenBank accession nos: BT094733, AK245399, U40979, AF165159, CX703216, BM188175, AW830581) encoding ACCases were identified as DEGs in the three contrast groups. All of them were down-regulated (log_2_ratio ≤ 1) in the three contrast groups except one gene (AK245356) encoding an ACCase was up-regulated (log_2_ratio = 1.4) at 35 DAF. Three co-expressed unigenes (GenBank accession nos: BM188175, CX703216, AW830581, EC6.3.4.14) that are associated with the transformation of BCCP to carboxybiotin-carboxyl-carrier protein were down-regulated during seed development.

The next enzyme in fatty acid pathway is *Fab*D, which catalyzes the transfer of malonyl-CoA to the holo acyl carrier protein (ACP), generating malonyl-ACP [21]. In the present study, the co-expressed unigene (AW34878) encoding *Fab*D was down-regulated during seed development.

*KAS*III (*Fab*H) catalyzes the subsequent condensation and transacylation of acetyl-CoA with malonyl-ACP and has a universal role in fatty acid biosynthesis. Transgenic *B. napus* seeds overexpressing *KAS*III driven by napin also contained lower oil levels compared to what was found in the wild-type [22]. In the present study, the co-expressed unigene (AF260565) encoding *KAS*III was significantly down-regulated in the fatty acid pathway with a log_2_ratio of −1.10, -2.05, and −2.09 in the three contrast groups. So compared to 15 DAF, the differentially expressed levels of the gene encoding *KAS*III at 35DAF, 55DAF, 65DAF negatively correlated to fatty acid synthesis during the seed development, which is consistent with previous research [23]. The same down-regulated patterns were also observed for *Fab*F (AF243183, CX702997, AK244605, AK285347), *Fab*Z (BT098415), *Fab*I (BI970856), *Fat*B (AK244101) and oleoyl-[acyl-carrier-protein] hydrolase (EC 3.1.2.14, AK244101).

In most plant tissues, Acyl-ACP thioesterase is the major determinant of chain length and level of saturated fatty acids [24]. It plays an important role by influencing the fatty acid composition of the produced oil and then mainly the ratio of 16 C to 18 C fatty acids and the level of saturated fatty acid [25]. Two distinct but related thioesterase gene classes exist in higher plants: *Fat*A is an acyl-specific thioesterase, with specificity for 18:1> > 18:0> > 16:0 fatty acids [26], which is considered an essential “housekeeping” enzyme in all plant cells; *Fat*B is a thioesterase, which shows specificity for 16:0 > 18:1 > 18:0 fatty acids [27]. In this study, Solexa sequencing analysis showed that the expression of gene (CX706542) encoding *Fat*A was unchanged at 35 DAF, but down-regulated at 55 DAF and 65 DAF with log_2_ratios of −1.7 and −1.5. While differentially co-expressed unigene (AK244101) encoding *Fat*B showed constant decreased expressed levels with log_2_ratios of −1.4, -2.4, -2.7, respectively. Therefore, the expression level variations of CX706542 and AK244101 would influence the 16 and 18 carbon fatty acids synthesis, which needs to be confirmed by experiment.

Soybean seeds contain three lipoxygenase isozymes; lipoxygenases 1, 2, and 3. Lipoxygenases (linoleate: oxygen oxidoreductase; EC 1.13.11.12) catalyze the oxidation of unsaturated fatty acids to produce conjugated unsaturated fatty acid hydroperoxides, which are converted to volatile compounds associated with undesirable flavors [28]. Eliminating this enzyme from seeds could lead to better quality soybean protein and oil products. Three co-expressed lipoxygenase genes (X67304, U50081, D13949) identified in this study were among those with the highest expression levels. To our knowledge, this is the first report of a close correlation between lipoxygenase expression and fatty acid accumulation.

The P450 family is a large and diverse group of isozymes that mediate a diverse array of oxidative reactions. The activities of most of these enzymes are associated with biosynthetic processes such as phenylpropanoid, terpenoid, and fatty acid biosyntheses. Ten alkane-inducible P450 genes from *Candida tropicalis* (ATCC20336), which were responsible for omega-hydroxylation of n-alkanes and fatty acids, were cloned [29]. In their research, these enzymes were believed to be at least in part limiting in the conversion of fatty acid to diacids, but their relative oxidative activity toward other fatty acids was not known. The two unigenes (AF022464, DQ340246) encoding soybean P450 monooxygenase were identified in this research. Both of them showed down-regulated expressions during seed development, which indicated that they are negatively correlated to the fatty acid accumulation. For the other differentially co-expressed unigenes shown in Table 2, there is little information available about their relationship with lipid accumulation. Their log_2_ratios indicate that they have significant functions in regulating soybean seed development. However, our results cannot validate a direct relationship between these unigenes and oil accumulation. This topic requires further research.

Because we are interested in oil production in soybeans, we selected the fatty acid biosynthesis pathway for deep analysis from among the 124 biochemical pathways identified by Solexa sequencing. Twenty four co-expressed unigenes in the fatty acid pathway, including 13 that overlapped with the unigenes identified in the GO analysis, showed significant correlations with fatty acid accumulation (Table 3).

The up-regulated genes that were significantly correlated with fatty acid accumulation included ACCase and lipoxygenase. The down-regulated genes that were significantly correlated with fatty acid accumulation included *Fab*D, *KAS* III, *Fab*F, *Fab*Z, *Fab*I, *Fat*B, *Fat*A, P450, and oleoyl-[acyl-carrier-protein] hydrolase genes. *Fab*G genes were both up- and down-regulated during seed development.

The analysis of the genes involved in the fatty acid biosynthetic pathway provides a basis to identify key regulatory processes controlling oil accumulation in soybean. However, biosynthetic pathways involve the cooperation of multiple genes. It is difficult to increase seed oil content by overexpressing a single gene. The large-scale characterization of unigenes described in this study shows comprehensive correlations between DEGs and fatty acid accumulation in soybean.

## Conclusion

In this study, 11592, 16594, and 16255 differentially expressed unigenes were identified at 35, 55, and 65 days after flowering of soybean, respectively. Gene Ontology analyses detected 113 co-expressed unigenes associated with lipid biosynthesis. Of these, 15 showed significant changes in expression levels (log_2_fold values ≥ 1) during seed development. Pathway analysis revealed 24 co-expressed transcripts involved in lipid biosynthesis and fatty acid biosynthesis pathways. These results provide a comprehensive molecular biology background for research on soybean seed development, particularly with respect to the process of oil accumulation. All of the genes identified in our research have significance for breeding soybeans with increased oil contents.

## Abbreviations

ACCase: acetyl CoA carboxylase; ACP: acyl carrier protein; BC: biotin carboxylase; BCCP: biotin carboxyl carrier protein; DAF: days after flowering; DE: differential expression; DEGs: differentially expressed genes; ER: endoplasmic reticulum; *Fab*A: 3-hydroxydecanoyl-[acyl-carrier-protein] dehydratase; *Fab*B: *Fab*F, *Fab*H or *KAS*III: 3-oxoacyl-[acyl-carrier-protein] synthase I, II, III; *Fab*D or *MCAT*: [acyl-carrier-protein] S-malonyltransferase; *Fab*G: 3-oxoacyl-[acyl-carrier protein] reductase; *Fab*I: *Fab*K, *Fab*L: enoyl-[acyl-carrier protein] reductase I, II, III; *Fab*Z: 3R-hydroxymyristoyl ACP dehydrase; *FAS*N: Fas: fatty acid synthase; *FAS*1: 2: fatty acid synthase subunit beta, alpha; *Fat*A: *Fat*B: fatty acyl-ACP thioesterase A, B; FDR: false discovery rate; MdcH: malonate decarboxylase epsilon subunit; RPKM: *reads*/Kb/million; TAG: triacylglycerol; TL: total lipids.

## Authors’ contributions

HC, FWW, YYD, NW, and YPS performed data analysis. HC, HYL wrote the manuscript. HYL and GC conceived the study. HC, HLY, YYJ, XYZ and YLL prepared the samples. HC, XYL, LL and XDF participated in the qRT-PCR verification. All the authors approved the final manuscript.

## Supplementary Material

Additional file 1 ** Table S1.** Differentially expressed genes between 35 DAF and 15 DAF (FDR ≤0.001 and |log_2_ratio| ≥1). Click here for file

Additional file 2 ** Table S2.** Differentially expressed genes between 55 DAF and 15 DAF (FDR ≤0.001 and |log_2_ratio| ≥1). Click here for file

Additional file 3 ** Table S3.** Differentially expressed genes between 65 DAF and 15 DAF (FDR ≤0.001 and |log_2_ratio| ≥1). Click here for file

Additional file 4 **Table S4.** Details of primers used for qRT-PCR. Click here for file

Additional file 5 ** Figure S1.** Scatter plot of differentially expressed genes in seeds harvested at 35 DAF, 55 DAF, and 65 DAF, compared with 15 DAF. (a). Scatter plot of differentially expressed genes between 35 DAF and 15 DAF. (b). Scatter plot of differentially expressed genes between 55 DAF and 15 DAF. (c). Scatter plot of differentially expressed genes between 65 DAF and 15 DAF. TPM = Transcript per million (normalized expression level of genes). Click here for file

Additional file 6 ** Figure S2.** Confirmation of differential gene expression by qRT-PCR. Click here for file
