# Supplementary material for: Sequence mining and transcript profiling to explore differentially expressed genes associated with lipid biosynthesis during soybean seed development
Source: BMC Plant Biol. 2012 Jul 31;12:122. doi: 10.1186/1471-2229-12-122 (PMC3490753; doi:10.1186/1471-2229-12-122)
Supplement: Additional file 4 — Table S4. Details of primers used for qRT-PCR. [file 1471-2229-12-122-S4.doc]

Supplementary Table A

**Real-time PCR confirmation of differential gene expression**

| GeneID | Target  Size(bp) | Solexa sequencing log2Ratio | Real-time PCR log2Ratio | Forward primer | Reverse primer | Sequence description |
| --- | --- | --- | --- | --- | --- | --- |
| 1794171 | 120 | 9.2494075 | 7.654869 | 5 ‘ GGAACTGGAGAACGCAAAGA 3’ | 5 ‘ AAAGGTGTCATTTCCTCCAAG 3’ | Glycine max lipoxygenase-3 mRNA (gb|U50081) |
| 255645838 | 145 | 8.398260393 | 8.0235687 | 5’ TTTGGATGGAGGACTTGGC 3’ | 5’ CAAAGCCTGCTGAGATGATTC 3’ | Fatty acid synthase activity (gb|BT098182) |
| 255635847 | 182 | 6.677823041 | 6.912386 | 5’ GTGACTCTGGGATTGGACG 3’ | 5’ CGTCGTAACCCAAATCAGC 3’ | Fatty acid synthase activity (gb|BT093926) |
| 210145849 | 124 | 5.286190724 | 5.46871368 | 5’ CGCCCTCTTCCATTTACCA 3’ | 5’ AGAGTTCCAGCCAGGGTCA 3’ | Lipid particle (gb|AK244540) |
| 18674 | 147 | 10.24842522 | 9.135697 | 5’ CTGGTGTAAATCCCTGCGTAA 3’ | 5’ TACCAAGTGCCTCGTCCATT 3’ | Glycine max mRNA for lipoxygenase 1 (gb|X67304) |
| 505137 | 127 | 8.72134321 | 7.025698 | 5’ AGATGGTTGCGGGTGTAAAT 3’ | 5’ GGGCATCTGCTGTTATCTTAC 3’ | Glycine max lox2 mRNA for lipoxygenase-2 (gb|D13949) |
| 18542 | 128 | 7.443814514 | 7.44356824 | 5’ TCCTGGTGGACCTCAATGG 3’ | 5’ ACTGAGGCATTGGTGGGTG 3’ | Soybean Bg gene for basic 7S globulin (gb|X16469) |
| 1199562 | 103 | 9.686158 | 9.5238436 | 5’ TAACCACGAAGAAGAGGCAAA 3’ | 5’ TAAACGATGAGAATGGGGTGA 3’ | Glycine max 34 kDa maturing seed vacuolar thiol protease mRNA  (gb|J05560) |
| 5230725 | 160 | 2.933911 | 3.1456233 | 5’ TCATTGGGCACCTTCACCT 3’ | 5’ TCGTGGGTTTGGAGGAAGA 3’ | Glycine max cytochrome P450 H2O2-dependent urate-degrading  peroxidase (PP1) mRNA (gb|AF089850) |
| 255641387 | 142 | 5.343235374 | 4.865473 | 5’ GAGGAAGAAGGTGATGGGACT 3’ | 5’ CTCCCATTTCAGCCTTTCG 3’ | Sulfotransferase activity (gb|BT096769) |
| 255637220 | 130 | 5.982628888 | 5.737554 | 5’ CCAAACCAGCATCAAGGAAA 3’ | 5’ ACCTTTGGCTGCTGAAGATTA 3’ | Water transmembrane transporter activity (gb|BT094631) |
| 42723003 | 138 | 7.414982533 | 8.55794563 | 5’ CCACCTTTCAAATGTAATCCAGT 3’ | 5’ AGAAAAGAACCCGTGAAGTGAG 3’ | Hydrolase activity (gb|CK768902) |
