# Supplementary figures and images for: Sequence mining and transcript profiling to explore differentially expressed genes associated with lipid biosynthesis during soybean seed development
Source: BMC Plant Biol. 2012 Jul 31;12:122. doi: 10.1186/1471-2229-12-122 (PMC3490753; doi:10.1186/1471-2229-12-122)

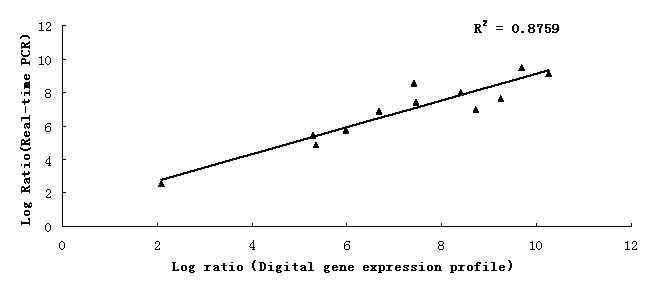

Supplement: Additional file 5 — Figure S1. Scatter plot of differentially expressed genes in seeds harvested at 35 DAF, 55 DAF, and 65 DAF, compared with 15 DAF. (a). Scatter plot of differentially expressed genes between 35 DAF and 15 DAF. (b). Scatter plot of differentially expressed genes between 55 DAF and 15 DAF. (c). Scatter plot of differentially expressed genes between 65 DAF and 15 DAF. TPM = Transcript per million (normalized expression level of genes). [file 1471-2229-12-122-S5.tiff]

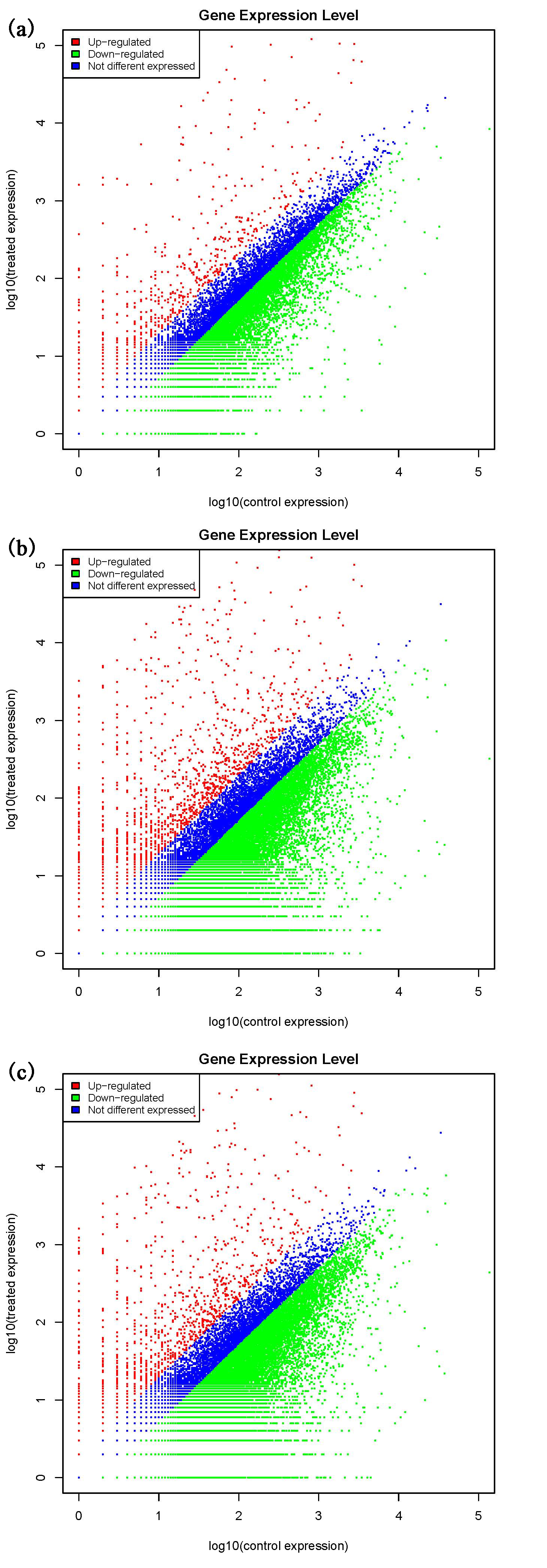

Supplement: Additional file 6 — Figure S2. Confirmation of differential gene expression by qRT-PCR. [file 1471-2229-12-122-S6.tiff]
